# Biochar affects carbon composition and stability in soil: a combined spectroscopy-microscopy study

**DOI:** 10.1038/srep25127

**Published:** 2016-04-26

**Authors:** Maria C. Hernandez-Soriano, Bart Kerré, Peter M. Kopittke, Benjamin Horemans, Erik Smolders

**Affiliations:** 1 Department of Earth and Environmental Science, KU Leuven, Kasteelpark Arenberg 20, B-3001 Heverlee, Belgium; 2 The University of Queensland, School of Agriculture and Food Sciences, St. Lucia, Queensland, 4072, Australia

## Abstract

The use of biochar can contribute to carbon (C) storage in soil. Upon addition of biochar, there is a spatial reorganization of C within soil particles, but the mechanisms remain unclear. Here, we used Fourier transformed infrared-microscopy and confocal laser scanning microscopy to examine this reorganization. A silty-loam soil was amended with three different organic residues and with the biochar produced from these residues and incubated for 237 d. Soil respiration was lower in biochar-amended soils than in residue-amended soils. Fluorescence analysis of the dissolved organic matter revealed that biochar application increased a humic-like fluorescent component, likely associated with biochar-C in solution. The combined spectroscopy-microscopy approach revealed the accumulation of aromatic-C in discrete spots in the solid-phase of microaggregates and its co-localization with clay minerals for soil amended with raw residue or biochar.The co-localization of aromatic-C:polysaccharides-C was consistently reduced upon biochar application. We conclude that reduced C metabolism is an important mechanism for C stabilization in biochar-amended soils.

The benefits of biochar application for carbon (C) sequestration and soil productivity have been examined extensively over the last decade. According to the review of Spokas *et al*.^1^, 50% of studies reported an increase in crop yield following application of biochar. These positive effects of biochar are generally related to the improvement of critical soil properties such as nutrient availability^2^,^3^,^4^, microbial activity^5^,^6^ and C stocks^4^,^7^,^8^. Given that biochar provides a pool of C that undergoes minimal microbial degradation^9^,^10^, the accumulation of this persistent pool of C within the soil can improve soil structure, water holding capacity and nutrient cycling^11^,^12^. In addition, biochar can have a protective effect for other sources of C within the soil^7^,^8^,^13^ and has been reported to decrease the mineralization rate of both native soil organic carbon (SOC) and fresh inputs of C such as raw residues^8^,^13^,^14^. This protective effect has been generally attributed to adsorption processes^15^,^16^ and is related to the composition and physicochemical properties of the biochar^5^,^17^, with these properties influenced by the choice of feedstock material and pyrolysis temperature^18^,^19^. However, adsorption of C on biochar surfaces can only partially explain this protective role and other mechanisms need to be examined. Particularly, the characterization of C distribution among functional pools is likely to provide new insights into the effects of biochar^20^,^21^,^22^, from molecular to field scale. The presence of biochar within microaggregates (<250 μm) has been reported in long-term biochar-enriched soils^20^,^23^,^24^, as well as the enhanced accumulation of newly added C in that soil fraction^8^,^13^. Short-term studies (<2 years) suggest that freshly added biochar promotes soil aggregation^22^,^25^, but measurable incorporation of biochar in soil microaggregates by traditional macroscopic measurements might not be feasible over such time scales^25^,^26^.

Characterization of organic matter based on traditional Fourier transform infrared spectroscopy (FTIR) has demonstrated that intensities of distinct peaks can be related to accumulation of C and formation of microaggregates^27^. Using FTIR analysis, Kimetu & Lehmann^28^ reported an increase in aromatic-C within aggregates of a biochar-amended soil. However, the examination of the lateral distribution of microaggregate-associated forms of organic carbon (OC) at high resolution can contribute to an understanding of the mechanisms influencing C stabilization in soil by identifying the molecular organization of OC, physical protection in the soil particles and co-localization of C sources with microbial processes^29^,^30^. Currently, few studies have examined the distribution of C forms in soil particles using spectroscopic techniques^29^,^30^,^31^,^32^, with these studies obtaining spectral maps of C speciation for selected areas of microaggregates isolated from C-rich forest soils. To the best of our knowledge, our study is the first to using FTIR-microscopy to map the distribution of C forms in entire microaggregates from a C-poor soil, amended with biochar or with the corresponding raw residues. Moreover, we combine the information obtained from the spectral maps of microaggregates with the characterization of dissolved organic matter (DOM) composition in the soluble phase by fluorescence spectrofluorometry. Excitation−emission matrix (EEM) obtained by fluorescence spectrofluorometry was used to examined the presence of common pools of fluorophores in DOM^33^,^34^,^35^, as well as indices related to source, redox state and biological reactivity of DOM^34^,^36^. Thus, fluorescent components identified in the DOM reflect changes in chemical properties of DOM associated to changes in the soil environment^35^,^37^ and can be directly linked to C cycling and metabolism in soil^38^.

The objective of the present study was to examine the mechanisms whereby biochar can potentially enhance C storage in soil. Of particular interest was the (i) characterization of molecular composition of organic matter in soil functional pools and (ii) lateral distribution of C forms in soil microaggregates. Experiments were established to provide a spectroscopic assessment of the impact of biochar on the turnover and build-up of C in soil. We have combined the measurement of C respiration rates in soil amended with biochar derived from different feedstocks with (i) characterization of the composition of dissolved organic carbon (DOC), and (ii) analysis of the composition and lateral distribution of OC in soil microaggregates. The effects of biochar were contrasted with those of the addition of raw residues, thereby allowing better discrimination of the effects of biochar.

## Results

The C:N ratios of the biochar products were similar to their corresponding feedstock material, although as expected the OC content was ca. 30% higher (P_B_) for the biochar (Table 1). The SA values of biochar determined using N_2_ isotherms (2.55 to 39.7 m^2 ^g^−1^) were comparatively low (for example, Downie *et al*.^39^ reported values up to 1500 m^2 ^g^−1^), presumably due to the obstruction of the nanopores^16^ – these pores (<2 nm) contributing most of the surface area of biochars. Indeed, CO_2_ provided higher SA values than N_2_ because it is less kinetically restricted at the temperature where its isotherm is constructed (273 K, c.f. 77 K for N_2_). Of the three biochar products, biochar derived from maize residue had the lowest SA irrespective of the method of measurement.

### Carbon mineralization

**Figure 1:**
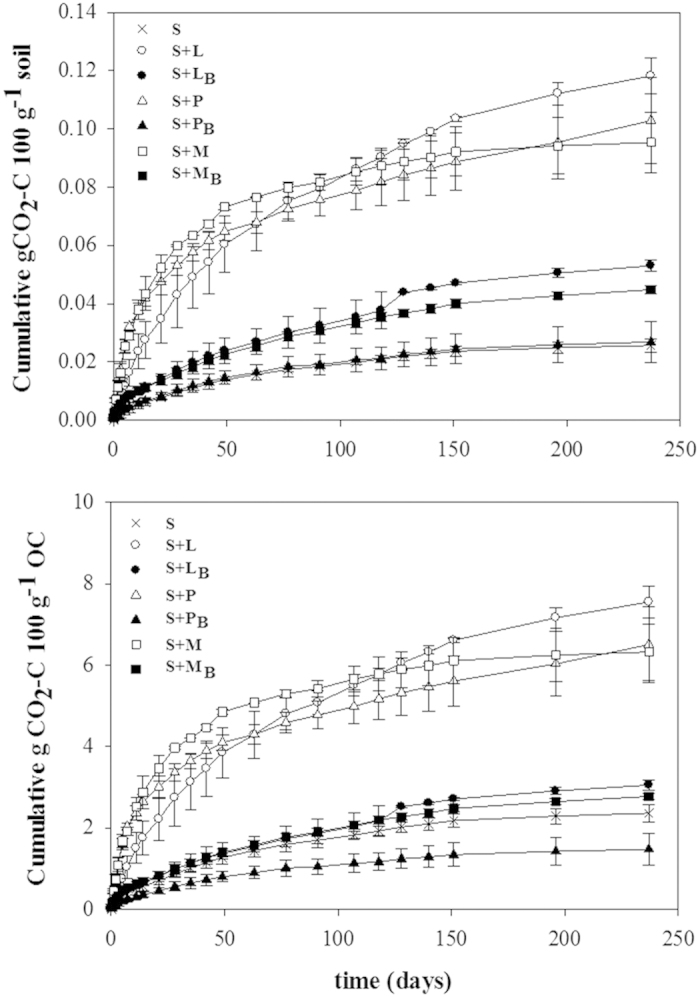
Carbon mineralization. Cumulative respiration of organic carbon (OC) relative to total soil weight or total OC in soil after 237 d of incubation for non-amended soil (S) and soil amended with leaf litter (S + L), peanut shell (S + P), maize residue (S + M) or biochar (B) derived from those materials (S + L_B_, S + P_B_ or S + M_B_). Data points are average values and bars are standard deviations (n = 3).

Carbon mineralization rates in soils amended with raw residues or biochar are summarized in Fig. 1, expressed as mineralization relative to soil weight (Fig. 1a) or normalized to total content of OC, i.e. native SOC plus C added with the raw residue or biochar material (Fig. 1b). The application of raw residues increased (p < 0.05) respiration rates about five-fold compared to non-amended soil (Fig. 1a). However, the increase in respiration rate following the addition of biochar was less (p < 0.05) pronounced, with respiration rate increasing either ca. two-fold for soil added with biochar prepared from maize residue or leaf litter or not at all for the addition of peanut shell-derived biochar. For the application of raw residues (S + M, S + P, S + L), ca. 6.0–7.5% of the initial OC was respired after 237 d of incubation, with no significant difference between the three sources (Fig. 1b). These cumulative respired fractions of OC in biochar amended soils were 1.5% for soil added with peanut shell-derived biochar, lower than for non-amended soil (2.4%) or soil added with biochar prepared from maize residue or leaf litter (2.8‒3.0%) (Fig. 1b).

### Double-exponential decay model and priming effects

The application of the three raw residues yielded similar cumulative emissions of CO_2_ (Fig. 1) but we observed differences in the distribution and decomposition rates of C among the labile and refractory pools (Table 2). Overall, the pool of refractory C (Refractory, i.e. slow mineralization) was larger than the pool of labile C (Labile, i.e. rapid mineralization) for all treatments (Table 2). The addition of raw residues increased the labile pool about 7- to 12-fold (p < 0.05) compared to non-amended soil (S), whilst biochar application had only a marginal effect (p > 0.05). The addition of raw residues increased (p < 0.05) the refractory pool up to three-fold compared to non-amended soil, while biochar addition resulted in either an increase (soil added with biochar prepared from maize residue or leaf litter) or decrease (soil added with peanut shell-derived biochar) of the refractory pool (p < 0.05). The application of the three biochar products yielded comparable amounts of C for the labile pool (p > 0.05) but the application of peanut shell-derived biochar yielded a significantly lower (p < 0.05) amount of C in the refractory pool compared to soil added with biochar obtained from maize residue or leaf litter.

The values of the mineralization constants estimated for the labile pool (k_L_) were not significantly affected by application of the raw residues or biochar compared to non-amended soil. However, the mineralization rate of the refractory pool (k_R_) significantly decreased with the application of leaf litter and peanut shell or the biochar prepared from these residues while maize residue increased k_R_ (Table 2). The k_L_ values were higher (p < 0.05) for soil amended with biochar compared to soil added with raw amendments (except for peanut shell-derived biochar) but that faster degradation of labile C in biochar treated soils is associated with distinct smaller concentrations of the labile C compared to those soils with raw amendments. Differences in k_R_ between the addition of raw residue and the derived biochar were negligible except for the addition of maize residue.

### Characteristics and composition of dissolved organic matter

The pore water was isolated from each soil at the end of the incubation and the DOC and UV absorbance at 254 nm were measured (Table 3). For soil amended with the raw residues, the DOC concentration was 22% higher (p < 0.05) for soil added with maize residue relative to non-amended soil after 237 d of incubation while for soil added with peanut shell and leaf litter the DOC was 9–30% lower than for non-amended soil (p < 0.05). For biochar-amended soils, the DOC was 45% lower (p < 0.05) for soil added with peanut shell-derived biochar after 237 d of incubation relative to the non-amended soil, while addition of biochar prepared from leaf litter or maize residue did not have a significant effect on DOC concentration (p > 0.05).

**Figure 2:**
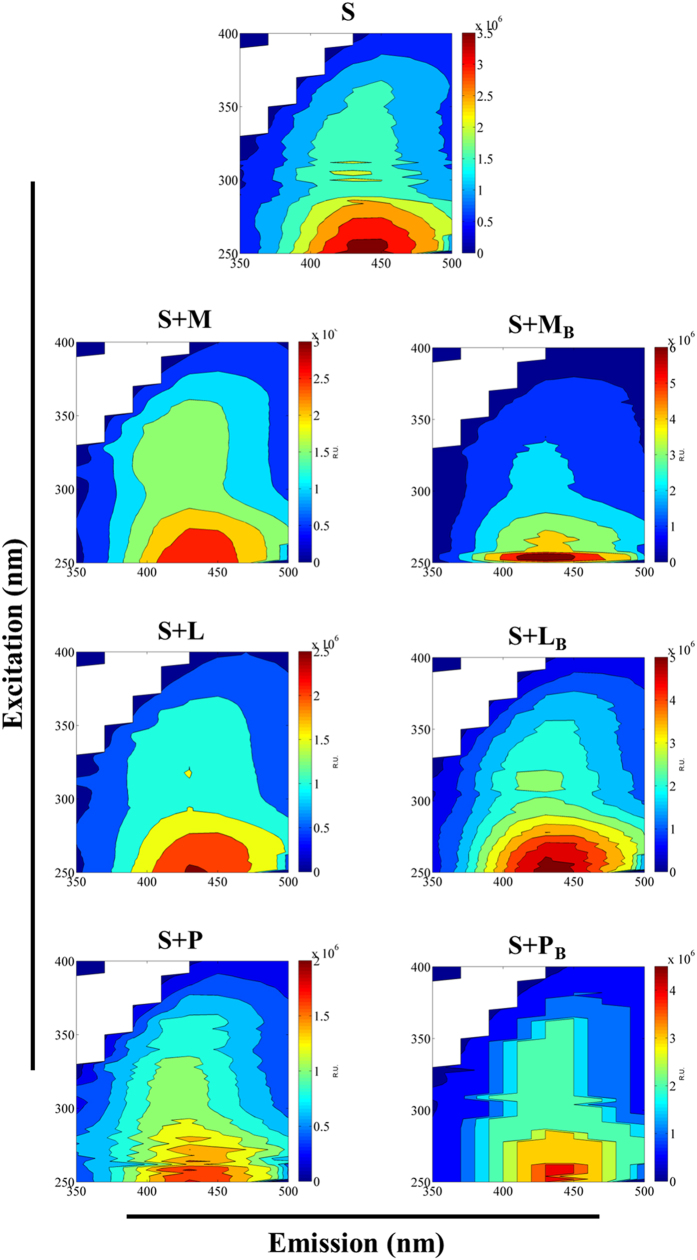
Fluorescent components of DOM. Fluorescent components identified by parallel factor analysis (PARAFAC) based on excitation (Ex.)- emission (Em.) matrices of dissolved organic matter from the different treatments . The components were assigned to an oxidized quinone-like (component 1) and a humic-like (component 2) component.

Excitation-emission matrices were obtained for the DOC isolated from the incubation assays by spectrofluorometric analysis (Supplementary Fig. S1) from which the humification index (HIX), freshness index (β:α), fluorescence intensity (FI), redox index (RI) were calculated (Table 3). The HIX significantly increased for the application of maize residue and biochar derived from this residue compared to non-amended soil but decreased for the addition of peanut shell (Table 3). The freshness index values (β:α) were consistently <1 across all treatments and represented an enrichment of DOC in decomposed material^36^ after 237 d of incubation (Table 3). This is consistent with values estimated for the FI (>2 for all the assays), which indicated that DOC is mostly derived from microbial activity^36^. The redox index values (RI) were generally low, this being indicative of a prevalence of oxidized compounds in the DOC, but significantly increased in soil added with biochar prepared from maize residue or leaf litter. The soil added with leaf litter and peanut shell maintained a ratio 2:1 for the two fluorescent components (Fig. 2) identified in the DOC (Table 3), while for the addition of maize residue the ratio decreased to 1:1. The components corresponded to an oxidized quinone-like (FC1) and a humic-like (FC2) components according to established models described elsewhere^33^,^34^,^40^. Soil amendment with biochar consistently increased the presence of the humic-like component and reduced the occurrence of the quinone-like component below 15% (biochar prepared from leaf litter or maize residue).

### Carbon composition and lateral distribution in soil aggregates

**Figure 3:**
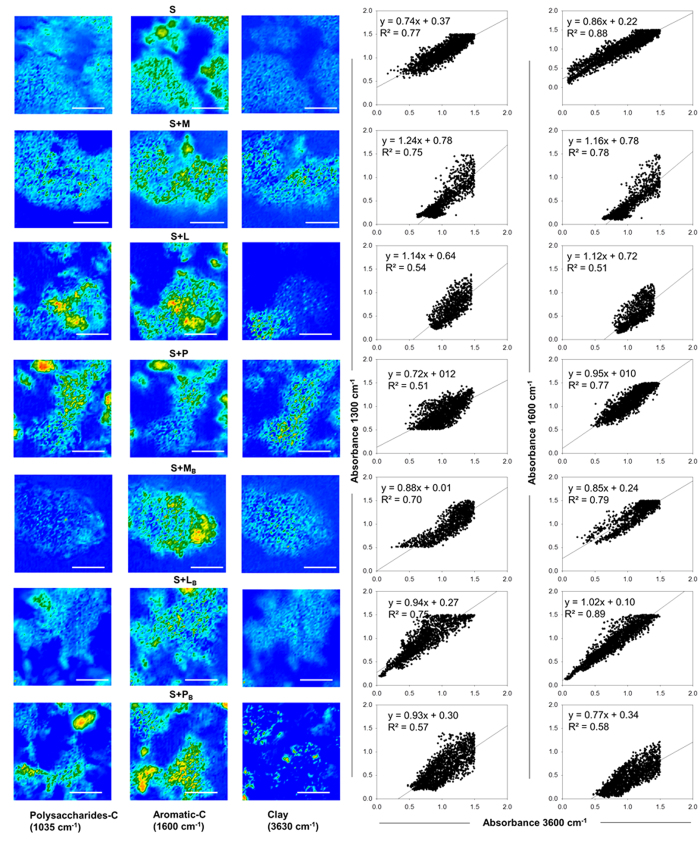
Maps of C speciation. Distribution of polysaccharides-C (1035 cm^−1^), aromatic-C (1600 cm^−1^) and mineral-OH (3630 cm^−1^) in microaggregates (<250 μm) isolated from non-amended soil (S) and soil amended with maize residue (S + M), leaf litter (S + L), peanut shell (S + P) or biochar (B) derived from those materials (S + M_B_, S + L_B_ or S + P_B_). Microaggregates were analysed by Fourier transform infrared spectroscopy (FTIR) coupled with optical microscopy. The images were obtained by combining 32 scans at a spectral resolution of 1 cm^−1^, lateral resolution 5 μm. Bars: 50 μm. Regression analyses were performed using the collection of spectra obtained for each map (i.e. one spectra per pixel).

We isolated intact microaggregates from the different assays after the long-incubation period and analysed the OC present in the particles by FTIR-microscopy. This novel technique provides a fingerprint of the main molecular structures present in the OC (Supplementary Fig. S2) while mapping the lateral distribution of the compounds identified in the microaggregate structure (Fig. 3).

The most relevant molecular features identified in the average spectra (Supplementary Fig. S2) corresponded to the presence of mineral-OH (3630 cm^−1^), polysaccharides (1035 cm^−1^), aliphatic compounds (2800‒2900 cm^−1^) and aromatic-C (1600 cm^−1^)^30^,^41^. The absorbance at 3630 cm^−1^ corresponds to stretching O-H in clays. Absorbance at 1600 cm^−1^ is associated to stretching modes of aromatics with multiple OH substitutions and has been previously identified in soils amended with biochar^41^. The presence of overtones in the range 1700‒2000 cm^−1^ indicated the presence of substituted phenyl rings. Signals around 700‒800 cm^−1^ were assigned to quartz^41^. The average spectra obtained for soil that had been amended with raw materials presented a signal at 1265 cm^−1^ assigned to carboxylates and amides that was not identified in non-amended soil. A slight increase in absorbance of the signal assigned to aliphatic carbon (2800‒2900 cm^−1^) was also observed in soil added with maize residue likely related to an increase in the presence of polysaccharides in the microaggregate. In contrast, for soil amended with biochar, the signal at 1265 cm^−1^ (assigned to carboxylates and amides) was reduced compared to soil added with raw materials. The spectra obtained for soil added with peanut shell-derived biochar presented a higher absorbance in the signal assigned to aliphatic C.

Second, after examination of the average spectra (Supplementary Fig. S2), the lateral distribution of mineral-OH (3630 cm^−1^), polysaccharides-C (1035 cm^−1^) and aromatic-C (1600 cm^−1^) in microaggregates isolated from non-amended soil or soil added with raw residue or residue-derived biochar were examined in the spectral maps (Fig. 3). The red-green-blue (RGB) composites obtained from the spectral maps (see Supplementary Fig. S3) provide a visual aid to recognize the simultaneous distribution of different functionalities in the aggregate. Mapping the FTIR signal assigned to clay-OH displayed a uniform distribution of this functionality across aggregates except for soil amended with leaf litter (S + L), peanut (S + P) or peanut shell-derived biochar (S + P_B_), which presented accumulation of clay in specific locations in the aggregate. This difference was accompanied by a significant decrease (p < 0.05) in the strength of the relationship (R^2^) of clay:polysaccharides-C for soil amended with leaf litter (S + L), peanut (S + P) or peanut shell-derived biochar (S + P_B_) compared to non-amended soil (Fig. 3 and Supplementary Table S1). Also, the relationship clay:aromatic-C significantly decreased (p < 0.05) for soil amended with leaf litter or peanut shell-derived biochar compared to the other treatments. Indeed, areas highly enriched in aromatic-C were observed in the maps corresponding to soil amended with leaf litter or biochar prepared from peanut shell or from maize residue (Fig. 3 and Supplementary Fig. S3).

Polysaccharides were generally co-localized with the aromatic-C across the different treatments (Fig. 3; Supplementary Table S1 and Supplementary Fig. S3). Presumably, aromatic-C in the microaggregates is largely derived from the accumulation of C metabolism products, with microbial activity being also responsible for the deposit of polysaccharides-C. The regression analysis (Supplementary Table 1) showed a slight and significant decrease in the relationship aromatic-C:polysaccharides-C for soil amended with biochar (R^2^ = 0.83–0.85) compared to soil non-amended or amended with raw residues (R^2^ > 0.92), also displayed in the spectral maps (Fig. 3 and Supplementary Fig. S3).

**Figure 4:**
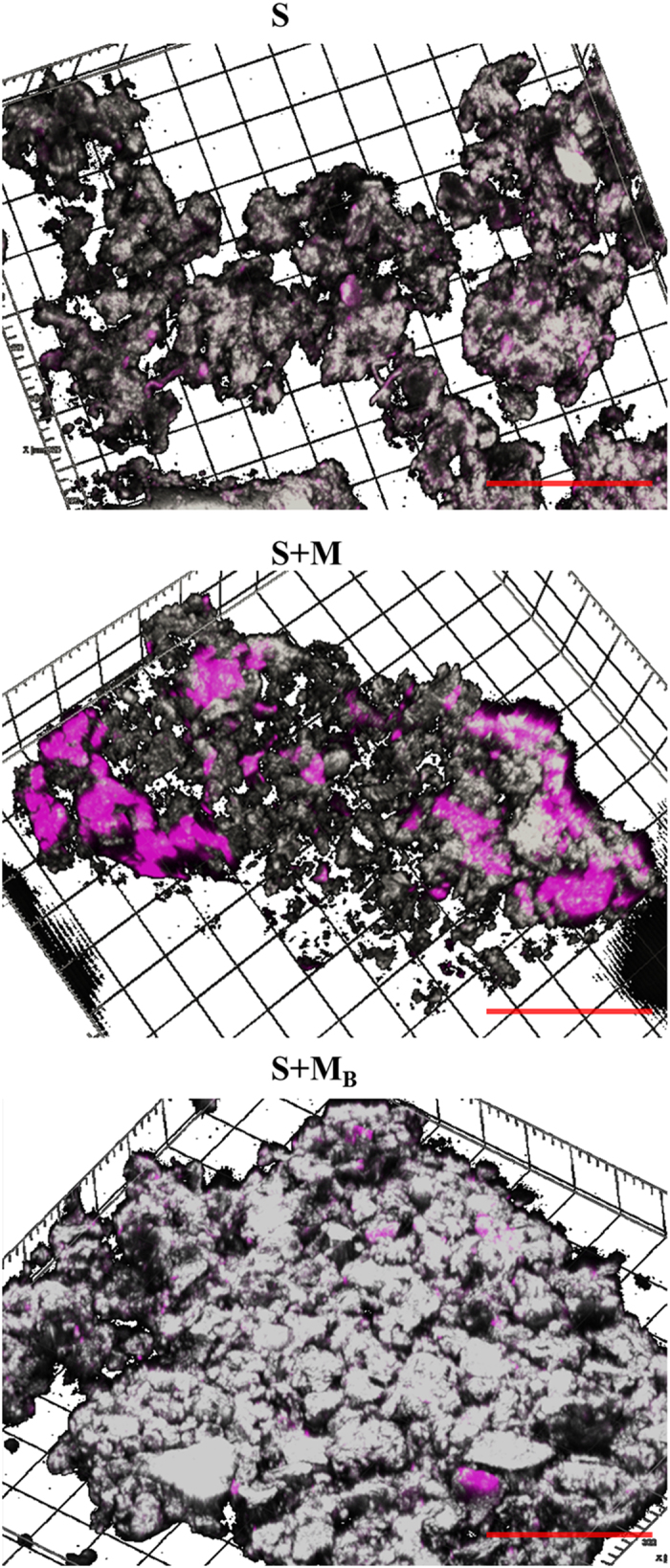
New insights via confocal imaging. Confocal laser scanning microscopy (CLSM) analysis visualizing autofluorescent polysaccharides (magenta) on soil aggregates from non-amended soil (S) and soil amended with maize residue (S + M), leaf litter (S + L), peanut shell (S + P) or biochar (B) derived from those materials (S + M_B_, S + L_B_ or S + P_B_). Excitation with a 375-nm laser, emission captured between 455 and 500 nm. Bars: 100 μm. Aggregate surfaces (grey) are visualized by capturing reflection of the 635 nm laser between 575–675 nm.

Distribution of OC in soil aggregates was further examined by CLSM (Fig. 4 and Supplementary Fig. S4). Areas of the aggregates that were identified as having preferential accumulation of OC enriched in autofluorescent molecules were visualized by excitation with a 375 nm laser. Emission in the range captured (455–500 nm) can be assigned to aromatic-C^42^. Single scans (Fig. 4) and the three-dimensional reconstructions (Supplementary Fig. S4) displayed a distribution of aromatic-C in the aggregates consistent with the mapping of the same compounds obtained by FTIR-microscopy. Hence, the aggregates from non-amended soil presented a homogeneous distribution of aromatic-C whereas aggregates from soil amended with raw residue or biochar presented discrete areas of selective enrichment in autofluorescent OC, this being comparable to the spots of high accumulation of aromatic-C observed in the FTIR maps (Fig. 3). Indeed, these deposits of aromatic-C were particularly pronounced for soil treated with leaf litter and biochar obtained from maize residue, as previously observed in the spectral maps.

## Discussion

Our study highlights the potential benefits of biochar application to soil for C stabilization and reduction of CO_2_ emissions compared to raw amendments^43^. For soil to which biochar was added, emissions of CO_2_ were ca. three-fold lower than from soils to which raw residues were added (Fig. 1). We also provide evidences that biochar influences the composition of DOM as well as the composition and distribution of OC in soil microaggregates – this being important for influencing C storage in soil^27^,^44^, associated biological processes^5^,^31^ and overall soil quality^21^.

Our results demonstrate that soil amended with biochar maintains C mineralization rates comparable to non-amended soil while significantly reducing CO_2_ emissions compared to the application of raw materials (Fig. 1)^19^,^23^,^45^. Moreover, we observed differences in the distribution and decomposition rate of C among the labile and refractory pools for the different treatments (Table 2), which were likely related to the properties of the amendments (Table 1)^45^,^46^. The variability in C partitioning and decomposition across treatments presumably reflects differences in C metabolism^6^,^12^,^22^, which might result in differences in the accumulation of C in soil functional pools^29^,^47^.

The decomposition of C in soil amended with maize residue resulted in higher accumulation of aromatic metabolic products in the soluble phase (DOM) after 237 d of incubation compared to soil added with leaf litter or peanut shell. Thus, addition of maize residue yielded a higher percentage of the humic-like component (FC2) in the DOM (Table 3, Fig. 2 and Supplementary Fig. S1) and a higher value of the humification index (HIX, Table 3) at the end of the 237 d incubation period. This variability in C processing following soil addition with different materials can be attributed to differences in the chemical composition of the C inputs and also influences C incorporation to soil organic matter^35^,^48^.

The mapping of the signal assigned to aromatic-C (1600 cm^−1^) in microaggregates from soil added with raw residues displayed the accumulation of these functions in discrete spots (Fig. 3), which were also visualized using CLSM (Fig. 4 and Supplementary Fig. S4). Indeed, we show for the first time the distribution of autofluorescent OC, corresponding to aromatic-C^42^, in single scans (Fig. 4) and three-dimensional reconstructions of microaggregates obtained by CLSM (Supplementary Fig. S4). These discrete deposits of aromatic-C are likely derived from C metabolism^29^,^31^ and can be linked to the enhanced C decomposition observed for the respiration assay (Fig. 1). Indeed, Vogel *et al*.^49^ recently demonstrated by Nano-scale secondary ion mass spectrometry (NanoSIMS) that C newly incorporated in soil particles is accumulated in discrete spots. Also, Wan *et al*.^32^ observed the accumulation of aromatic-C in distinct areas of spectral maps of soil particles (<43 μm) obtained using C NEXAFS.

Organo-mineral interactions and polysaccharides binding might have a substantial role in the stabilization of OC^31^,^50^ for soil added with maize residue and peanut shell. The aromatic-C spots were co-localized with the signal assigned to clay-OH and polysaccharides-C (Fig. 3, Supplementary Table 1). However, these mechanisms might not suffice to explain OC stabilization in soil added with leaf litter. The correlation aromatic-C:clay significantly decreased for soil added with leaf litter compared to non-amended soil or soil added with maize residue or peanut shell (Supplementary Table 1) whilst spectral maps (Fig. 3) and derived RGB composites (Supplementary Fig. S3) revealed a segregation of clay and aromatic-C and a substantial reduction in the occurrence of polysaccharides-C. Thus, additional mechanisms of C stabilization need to be considered for soil added with leaf litter, such as H-bonding, electrostatic binding and hydrophobic interactions^29^,^51^.

The biochar produced in the present study provided a source of OC that either maintained (peanut shell-derived biochar) or marginally but significantly increased (biochar prepared from leaf litter or maize residue) total soil respiration compared to non-amended soil (Fig. 1). This slight increase in respiration presumably represented a modest increase in the mineralizable (labile) C pool^44^ as estimated using the double exponential model (Fig. 1, Table 2). Previous studies have indicated that the release of CO_2_ adsorbed or precipitated as carbonates on the alkaline biochar surface during biochar production can contribute to initial rates of CO_2_ emission in biochar amended soil^52^.

The application of biochar obtained from leaf litter or maize residue to soil yielded similar respiration rates (Fig. 1, Table 2) and DOC concentrations (Table 3) after 237 d of incubation than non-amended soil. However, composition of the DOM was substantially affected by addition of biochar, which suggest relevant changes in C processing^35^. Indeed, previous research has proposed that changes in soil microbial communities might explain the influence of biochar in C metabolism^6^,^9^. Steinbeiss *et al*.^5^ observed that microbial groups in arable soil could promptly adapt to biochar degradation. The application of biochar likely modifies the soil habitat, providing new microhabitats for microbial activity of specific communities in its porous surface^6^. Besides, increase in nutrients bioavailability of P, Ca, Mg, K^53^, enhanced soil pH^12^ and the presence of biochar-associated labile volatiles compounds^54^ are additional factors that might affect microbial biomass and activity. Other authors have suggested that biochar might induce solubilisation of soil organic matter, which would indeed increase DOM aromaticity^55^. Here, we observed that biochar application to soil can alter the presence of fluorescent components in the DOM (Fig. 2 and Supplementary Fig. S1). Thus, biochar application increased the percentage of a humic-like component (2–3 times higher) for biochar amended soil compared to non-amended soil or soil added with raw residues, while the percentage of oxidized quinone component decreased 1.5–12 times (Table 3). These results might suggest that biochar application contributes to the composition of DOM with a pool of humic-like C. Indeed, Jamieson *et al*.^56^ described a strong contribution of humic-like components in the DOM from three different biochar leachates.

The microaggregates isolated from biochar-amended soil displayed similar accumulation of aromatic-C in discrete spots than described above for soil added with the raw residues (Figs 3 and 4). Also, the distribution of aromatic-C and clay were well correlated (Supplementary Table 1) for soil added with biochar obtained from maize residue or leaf litter. Thus, organo-mineral interactions might also be partially responsible for the accumulation of C in biochar amended soil, as previously discussed in the literature^12^,^44^. Indeed, the presence of mineral phases on biochar surfaces has been observed using scanning electron microscopy^57^ and the authors reported the formation of biochar-soil mineral complexes shortly after application of biochar to soil. However, the correlation aromatic-C: clay was significantly lower for soil added with peanut shell-derived biochar than for soil added with biochar prepared from maize residue or leaf litter (Supplementary Table 1). Interestingly, the application of the three biochar products significant decreased the correlation aromatic-C:polysaccharides-C compared to non-amended soil or soil added with the raw residues (Supplementary Table S1) presumably due to a reduction in C biological processing. Also, the distribution of polysaccharides displayed in Fig. 3 and the RGB composites (Supplementary Fig. S3) suggest a reduction on the presence of polysaccharides-C for soil added with biochar prepared from maize residue or leaf litter compared to non-amended soil or soil added with raw residues, but such effect is not observed for the addition of biochar produced from peanut shell. These results further support changes in C metabolism upon biochar application^5^,^9^ while also confirm that different biochar products have varied effects in C balance^18^,^39^.

Our results have further implications. Particularly, the results presented in this study support that: i) information obtained from spatial analysis of carbon species in soil particles can be linked to chemical and biological processes related to carbon stabilization in soil and ii) the fraction of carbon in the soluble phase and its composition might have a crucial role in the stabilization and processing of different sources of carbon in soil. These findings are consistent with some of the approaches recently discussed in the literature^58^. Thus, Lehmann and Kleber^58^ suggest that spatial data might be used for the development of models that can predict carbon dynamics in soil. Also, these authors discuss the relevance of carbon in the soluble phase in carbon processing.

Overall, the present study provides new evidences of the changes that occur in the composition of OC in two key functional pools, the DOC and the microaggregates, upon application of raw residues or biochar products. These results have important implications for C metabolism and storage in soil particles. Particularly relevant is the information obtained from the combination of spectroscopic techniques with microscopy. This approach has demonstrated that not only changes in functional groups but their distribution in the microaggregates are influenced by the presence of biochar in soil – this being indicative of changes in the physical protection and processing of C in soil. Nevertheless, our results also confirm a limited consistency of treatment effect for different biochar products and suggest that selection of feedstock material is critical for an effective application of biochar to soil.

## Methods

### Soil, raw amendments and biochar

Soil was collected from an agricultural soil in Belgium (50°52′42″N, 4°39′24″E). The soil is silt loam Haplic Luvisol (International Union of Soil Sciences, 2014). Initial analyses of the soil indicated: 0.79% OM, a cation exchange capacity (CEC) of 15 cmol_c_ kg^−1^, pH (10 mM CaCl_2_) of 7.1 and a texture of 35/53/12 (sand/silt/clay). The topsoil was sampled (0‒25 cm), sieved (<2 mm) and incubated for 7 d at 25 °C in darkness before addition of the raw and biochar materials.

Biochar samples were prepared from residues of maize (*Zea mays* L., leaves plus stalks), peanut (*Arachis hypogaea* L., shell) or London Plane (*Platanus hispanica*, dry leaf litter) in a muffle furnace according to the procedure described by Kookana^59^. Samples were prepared under oxygen-limiting conditions by using compactly closed crucibles. The furnace was programmed to reach 450 °C in 1 h and to hold this temperature for 2 h. The biochar samples were collected and cooled to room temperature. Mass recovery was determined by gravimetric analysis, being 30‒42% for all samples.

Total C content and C:N ratios were determined for the raw residues and derived biochar samples. Samples were bullet-ground, weighed in silver capsules, acidified with 20 μL of HCl 10% (w/w) to remove inorganic C and oven-dried overnight at 50 °C. Analyses where performed with a FlashEA 1112 HT elemental analyzer (Thermo Fisher Scientific, Bremen, Germany).

The surface area (SA) and pore size distribution were measured for the biochar samples by N_2_ (77 K), Brunauer-Emmett-Teller adsorption^60^, and CO_2_ (273 K) adsorption, used for pores <1.5 nm (Amstaetter *et al*., 2012; Hinz, 2001) on an Autosorb-1 (Quantachrome Instruments, Boynton Beach, FL, USA) after outgassing at 200 °C. Pore size distribution was calculated using the Grand-Canonical-Monte-Carlo method.

### Soil respiration

Seven treatments were prepared in triplicate by mixing soil (S) with maize residue (S + M), biochar obtained from maize residue (S + M_B_), dry leaf litter (S + L), biochar obtained from dry leaf litter (S + L_B_), peanut shell (S + P) or biochar prepared from peanut shell (S + P_B_), in addition to the preparation of a non-amended soil (S), giving a total of 21 experimental units. The different amendments were finely ground and added to samples of soil (20 g) at 1% (dry weight). The mixtures were thoroughly mixed and placed in 300 mL air-tight glass jars equipped with three-way valves allowing air sampling from the headspace. Moisture content (16% w/w) was adjusted with MilliQ water. The jars were closed and incubated in the darkness at 25 °C for 237 d. Two empty jars (blanks) were incubated in the same conditions.

The air from the headspace was sampled periodically with a 60 mL syringe and injected in a LI-COR CO_2_ infrared gas analyzer (LI-820). The gas stream passes through a Mg(ClO_4_)_2_ (Sercon, UK) absorptive water trap to remove water vapor from the air sample and the setup was equipped with a CO_2_ trap (Carbosorb, Sercon, UK) to allow flushing the system with CO_2_-free air in between measurements. Following measurement, the jars were left open for 5–10 min to ensure equilibration with atmospheric CO_2_. The soil moisture content was measured gravimetrically and corrected with milliQ water as necessary to maintain moisture at 16% (w/w). The CO_2_-values measured (ppm) were corrected by subtraction of the blanks and the amount of C respired calculated using the ideal gas equation. The pH of the soil was measured after 237 d of incubation for all the treatments.

### Mineralization kinetics

The kinetics of OC mineralization were described for the different assays by fitting a double-exponential model to the respiration data. The model assumes two pools of mineralizable OC^45^:


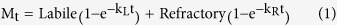


where M_t_ is the amount of mineralizable C (g C 100 g^−1^ SOC), Labile is the size of labile C pool (normalized per unit SOC), Refractory is the size of refractory C pool (normalized per unit SOC), t is the incubation time (d) and k_L_ and k_R_ are the first order mineralization rate constants for the Labile and Refractory pools of C (d^−1^) respectively. Model parameters were estimated from the best-fit model using SPSS v.17 software package. Non-linear regression using a Levenberg-Marquardt algorithm returned the best-fit parameters by minimizing the sum of squares of the residuals between measured and fitted values. The half-life of C in the soil (t_1/2_) of each treatment was calculated on the basis of k_R_: t_1/2_ = ln (2)/k_R_.

### Dissolved organic matter characterization by spectrofluorometry

Soil samples (100 g) were incubated (triplicate) for 237 d following the same experimental setup described for the respiration assays. In this study, the objective was not to align C mineralization with DOC concentration or characteristics but to examine differences in DOM composition at the end of the incubation period as endpoints for the long-term impact of the different treatments.

The DOM was examined in the soil solution, obtained from the soil samples after 237 d of incubation, through centrifugation (10 min at 3000 g) using the ‘double chamber’ method^61^. After centrifugation, the soil solution samples were immediately filtered (0.45-μm) and analyzed for DOC using a TOC-analyser (Analytical Sciences Thermalox). The UV-absorbance was measured with a UV-VIS spectrophotometer (Perkin-Elmer, Lambda 20, quartz cells) and the specific UV absorbance (SUVA, L g^−1 ^cm^−1^) was estimated.

Soil solution samples were diluted to obtain an absorbance reading at 254 nm below 0.2 prior to the collection of fluorescence spectra^62^. Fluorescence excitation-emission spectra (EEMs) were obtained for the pore water solutions using a JY HORIBA Fluorolog-3 spectrofluorometer with an excitation range set from 240 to 400 nm and an emission range set from 300 to 500 nm in 2 nm increments. Instrumental parameters were: excitation and emission slits, 5 nm; response time, 8 s; and scan speed, 1200 nm min^−1^. Spectra were collected using the software FluorEssence.

Parallel factor analysis (PARAFAC) on EEMs was performed in MATLAB R2013a using the DOMFluor toolbox (ver. 1.7; Feb. 2009) developed for MATLAB by Colin Stedmon (NERI, Aarhus University, Denmark)^63^,^64^. The EEM datasets were blank-subtracted and corrected for scattering. Two fluorescent components (FC1 and FC2) were identified by PARAFAC analysis for the set of assays studied by split half analysis and analysis of residuals and loadings and were related to components described elsewhere^33^,^36^. We also calculated the humification index (HIX) indicative of the extent of humification^65^, the β:α or freshness index (FrI) indicative of contribution of newer or decomposed material to DOM^66^, the fluorescence index (FI) as a proxy for DOM source (i.e., terrigenous versus microbially derived DOM)^67^ and the redox index (RI) as the ratio of reduced:oxidized compounds of DOM^68^.

### Carbon composition and distribution in aggregates by FTIR–microscopy

Microaggregates particles (200 μm) were isolated by wet sieving from the different incubation assays after 237 d incubation. The intact microaggregates were analyzed using a Fourier transform infrared spectrophotometer (Varian 620-IR IR microscope) coupled to a microscope (FTIR-microscope) using a KBr splitter and a liquid nitrogen cooled Focal Plane Array detector for spectrochemical imaging and a CCD camera. Spectral maps of aggregates were recorded in the mid-infrared range (4000–800 cm^−1^) combining 32 scans with a spectral resolution of 1 cm^−1^ and lateral resolution 5.5 μm. Three randomly selected microaggregates were analyzed per treatment.

Spectral maps were processed using the software Agilent Resolutions Pro. After normalization and automatic baseline correction, map profiles were created for peak heights at 3630, 1600 and 1035 cm^−1^, main FTIR bands of common soil components. The peak position at 3630 cm^−1^ corresponds to O–H groups of clays, at 1600 cm^−1^ to aromatic C, and at 1035 cm^−1^ to polysaccharide C^29^. Additionally, RGB composite images were obtained from the spectral maps using GIMP 2.8.0 (GNU Image Manipulation Program, 2012, USA).

The spectra collected for each map were imported into the Unscrambler X 10.3 (CAMO Software AS, 2014, Norway) and regression analysis were performed to explore the relationship between the amount of clay (absorbance at 3630 cm^−1^) and either polysaccharides C (absorbance at 1035 cm−1), aromatic C (absorbance at 1600 cm^−1^) or aliphatic C (absorbance at 2922 cm^−1^)^30^,^41^. The R^2^ coefficients and regression slopes were used as two different indices for the associations, the former indicating the residual variability around the association while the latter indicating the relative strength of association. Both R^2^ and slopes were compared between treatments by using the three independent aggregates, i.e. the R^2^ and slope were derived per replicate aggregate and then used as replicate parameters. The treatment effects on these parameters were then tested with ANOVA’s followed by Holm-Sidak test using SigmaPlot 12.3 (Systat Software, Inc., 2014, USA).

### Carbon distribution in aggregates by CLSM

Distribution of OC on soil microaggregates was further examined by confocal laser scanning microscopy (CLSM) using an Olympus IX81 inverted microscope equipped with an Olympus Fluoview FV1000 confocal scanning unit. Aromatic-C was visualized by excitation with a 375 nm laser and emission captured between 455–500 nm (bandpass filter) (magenta color)^42^. The microaggregates were visualized by illumination of the 635 nm laser and capturing the reflection between 575‒675 nm (greyscale). Image size was 1024 × 1024 pixels (pixel size 0.31 μm), corresponding to 317.3 μm × 317.3 μm (x and y direction). We acquired in-focus images of the microaggregates by optical sectioning. Scans were performed with 1 μm increments in the z direction. Surface profiling of the microaggregate was obtained by three-dimensional reconstruction of the z-series image stacks.

### Statistical analysis

ANOVA’s followed by Holm-Sidak tests were used to compare respiration rates, pH values and DOC characteristics between multiple treatments using SigmaPlot 12.3 (Systat Software, Inc., 2014, USA). For all statistical tests, a p < 0.05 level of significance was considered.

## Additional Information

**How to cite this article**: Hernandez-Soriano, M. C. *et al*. Biochar affects carbon composition and stability in soil: a combined spectroscopy-microscopy study. *Sci. Rep*. **6**, 25127; doi: 10.1038/srep25127 (2016).

## Supplementary Material

Supplementary Information

## Figures and Tables

**Table 1:** Properties of biochar and raw amendments.

	OC (%)	C:N ratio	Surface area (m^2^g^−1^)	
			N_2_	CO_2_
Maize residue (M)	40 ± 1^a^	20 ± 1^a^		
Leaf litter (L)	50 ± 1^b^	70 ± 4^b^		
Peanut shell (P)	50 ± 1^b^	40 ± 8^c^		
Biochar M (M_B_)	50 ± 1^b^	20 ± 1^a^	2.55	232
Biochar L (L_B_)	60 ± 2^c^	70 ± 4^b^	38.2	339
Biochar P (P_B_)	70 ± 1^d^	50 ± 1^c^	39.7	390

Total organic carbon (OC) and carbon (C):nitrogen (N) ratio of the raw amendments and the biochar samples (average and standard deviation of triplicates). The surface areas (m^2^g^−1^) of the biochar products were obtained from Brunauer-Emmett-Teller fit of the N_2_ adsorption isotherm at 77 K and Grand-Canonical-Monte-Carlo method of the CO_2_ adsorption isotherm at 273 K. Treatments not connected by the same letter are significantly different (p < 0.05) within the same column.

**Table 2:** Mineralization kinetics.

	Double-exponential decay model parameters					
	Labile (g C kg^−1^ OC)	k_L_ (d^−1^)	Refractory (g C kg^−1^ OC)	k_R_(d^−1^)	SEE^a^	R^2^
S	2.2 ± 0.4^a^	0.604 ± 0.378^ab^	23 ± 1^a^	0.012 ± 0.001^a^	1.061	0.983
S + M	26 ± 3^b^	0.132 ± 0.019^a^	38 ± 2^b^	0.017 ± 0.002^b^	2.017	0.992
S + L	15 ± 3^c^	0.081 ± 0.023^a^	74 ± 3^c^	0.008 ± 0.001^cd^	2.385	0.991
S + P	27 ± 2^b^	0.147 ± 0.024^a^	64 ± 6^d^	0.006 ± 0.002^d^	3.366	0.973
S + M_B_	3.6 ± 0.3^a^	0.546 ± 0.144^ab^	28 ± 1^e^	0.010 ± 0.001^ac^	0.772	0.993
S + L_B_	3.3 ± 0.4^a^	0.812 ± 0.401^b^	34 ± 2^be^	0.007 ± 0.001^cd^	1.289	0.983
S + P_B_	4.0 ± 0.8^a^	0.081 ± 0.021^a^	19 ± 1^f^	0.007 ± 0.001^cd^	1.500	0.997

Pool sizes and decay rates for the different treatments, using a double-exponential decay model. (Labile = size of labile C pool (g C 100 g^−1^ SOC), Refractory = size of refractory C pool (g C 100 g^−1^ SOC), k_L_ = first order mineralization rate constant for the labile pool (d^−1^), k_R_ = first order mineralization rate constant for the refractory pool (d^−1^). S = soil; L = leaf litter; P = peanut shell; M = maize residue; L_B_ = biochar derived from leaf litter; P_B_ = biochar derived from peanut shell; M_B_ = biochar derived from maize residue. Treatments not connected by the same letter are significantly different (p < 0.05) within the same column.

^a^SEE: standard error of estimates (Residual sum of squares/corrected sum of square).

**Table 3:** Characterization of DOM.

	pH	DOC (mg L^−1^)	SUVA (L g^−1 ^cm^−1^)	HIX	β:α	FI	RI	FC1	FC2
S	5.6 ± 0.3^a^	70 ± 3^a^	30 ± 2^a^	4.1 ± 0.2^ac^	0.60 ± 0.03^a^	2.4 ± 0.1^a^	0.07 ± 0.01^ad^	70 ± 4^a^	30 ± 1^a^
S + M	5.6 ± 0.3^a^	85 ± 4^b^	30 ± 2^a^	5.6 ± 0.3^b^	0.61 ± 0.03^a^	2.5 ± 0.1^a^	0.07 ± 0.01^ad^	60 ± 3^b^	40 ± 2^b^
S + L	5.9 ± 0.3^a^	50 ± 2^cd^	50 ± 2^b^	4.3 ± 0.2^ac^	0.53 ± 0.03^a^	2.3 ± 0.1^a^	0.04 ± 0.01^a^	90 ± 4^c^	25 ± 1^a^
S + P	5.6 ± 0.3^a^	60 ± 3^c^	30 ± 2^a^	3.4 ± 0.2^c^	0.64 ± 0.03^a^	2.5 ± 0.1^a^	0.03 ± 0.01^a^	80 ± 4^ac^	10 ± 1^c^
S + M_B_	6.3 ± 0.3^a^	70 ± 3^a^	30 ± 1^a^	8.7 ± 0.4^d^	0.52 ± 0.03^a^	2.3 ± 0.1^a^	0.16 ± 0.01^b^	10 ± 1^d^	80 ± 4^d^
S + L_B_	6.1 ± 0.3^a^	70 ± 4^a^	25 ± 1^a^	4.7 ± 0.2^ab^	0.61 ± 0.03^a^	2.3 ± 0.1^a^	0.22 ± 0.01^c^	10 ± 1^d^	95 ± 5^e^
S + P_B_	5.7 ± 0.3^a^	40 ± 2^d^	25 ± 1^a^	4.2 ± 0.2^a^	0.57 ± 0.03^a^	2.3 ± 0.1^a^	0.09 ± 0.01^d^	50 ± 2^b^	55 ± 3^f^

Soil (S) pH after 237 d incubation, dissolved organic carbon (DOC), specific UV absorbance (SUVA), humification index (HIX), freshness index (β:α), fluorescence intensity (FI), redox index (RI) and percentage of fluorescent components estimated after 237 d of soil incubation with the raw amendments (L = leaf litter; P = peanut shell; M = maize residue) or the biochar materials (L_B_ = biochar derived from leaf litter; P_B_ = biochar derived from peanut shell; M_B_ = biochar derived from maize residue). FC1 and FC2 are fluorescent components identified by PARAFAC analysis, presented as percentages. Data correspond to average and relative standard error of triplicates. Treatments not connected by the same letter are significantly different (p < 0.05) within the same column.
