# Supplementary material for: Biochar affects carbon composition and stability in soil: a combined spectroscopy-microscopy study
Source: Sci Rep. 2016 Apr 26;6:25127. doi: 10.1038/srep25127 (PMC4844975; doi:10.1038/srep25127)
Supplement: Supplementary Information [file srep25127-s1.pdf]

Supplementary Information

Biochar affects carbon composition and stability in soil: a combined spectroscopy-microscopy study

Maria C. Hernandez-Soriano[1,2,]*Bart Kerre[1], Peter M. Kopittke[2], Benjamin Horemans[1], Erik Smolders[1]

[1]*Department of Earth and Environmental Science, KU Leuven, Kasteelpark Arenberg 20, B-3001 Heverlee, Belgium*

[2]*The University of Queensland, School of Agriculture and Food Sciences, St. Lucia, Queensland, 4072, Australia*

*Corresponding author: Dr. Maria C. Hernandez-Soriano

e-mail: m.hernandezsoriano@uq.edu.au

Phone: +61 7 3365 2573

Fax: +61 7 3365 1177

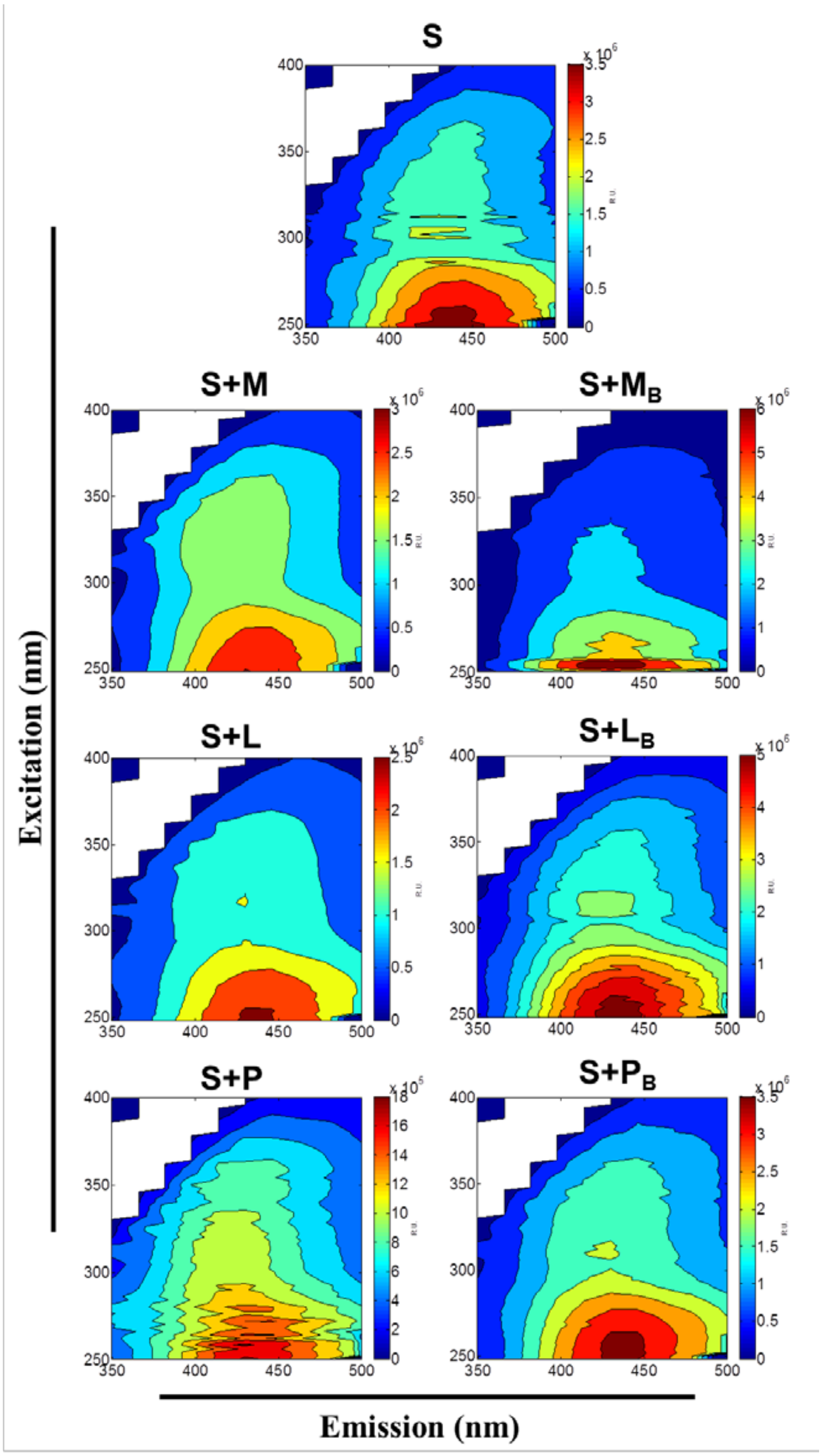

Figure S1. **Fluorescence excitation-emission matrices of DOM**. The EEMs obtained by spectrofluorometric analysis of soil solution from non-amended soil (S) and soil amended with maize residue (S+M), leaf litter (S+L), peanut shell (S+P) or biochar (B) derived from these materials (S+M_B, S+L_B, S+P_B).

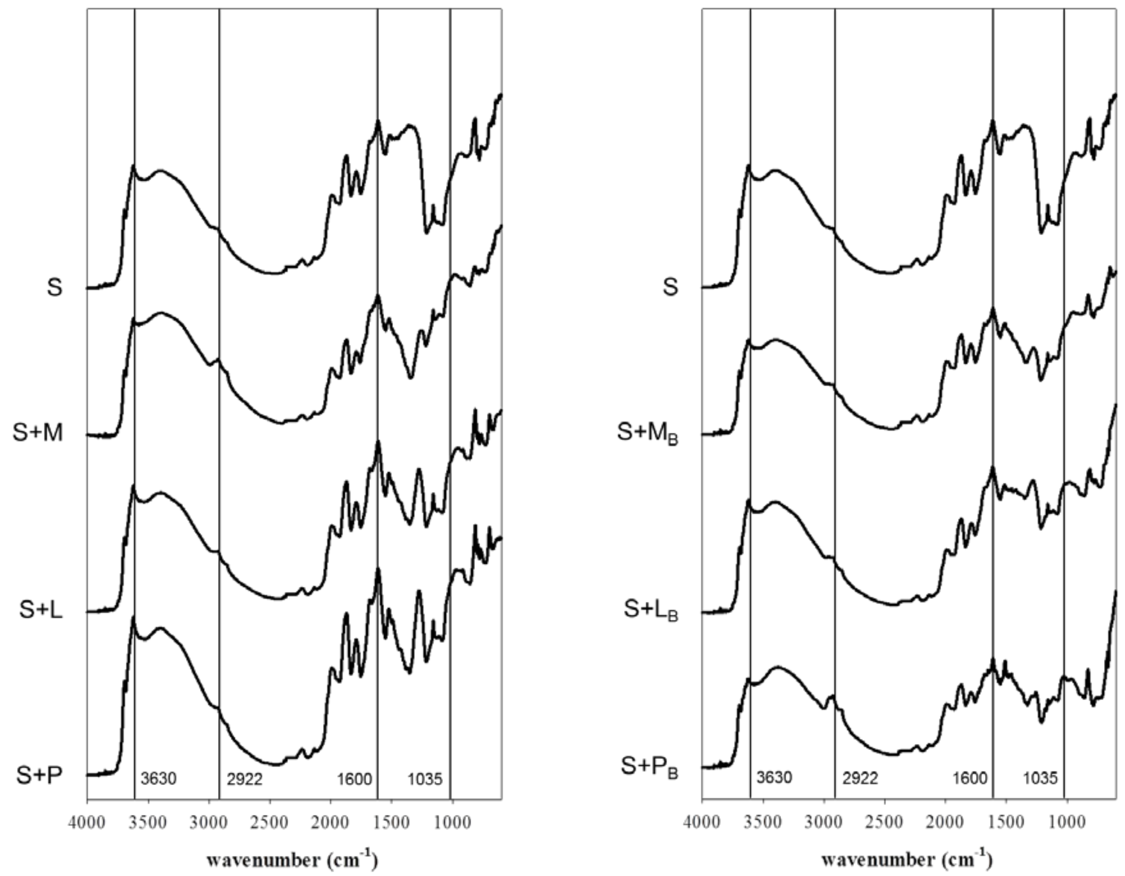

Figure S2. **FTIR spectra of soil microaggregates**. Average spectra (derived from the spectral maps) for microaggregates (<250 µm) isolated from non-amended soil (S) and soil amended with maize residue (S+M), leaf litter (S+L), peanut shell (S+P) or biochar (B) derived from those materials (S+M_B, S+L_B or S+P_B).

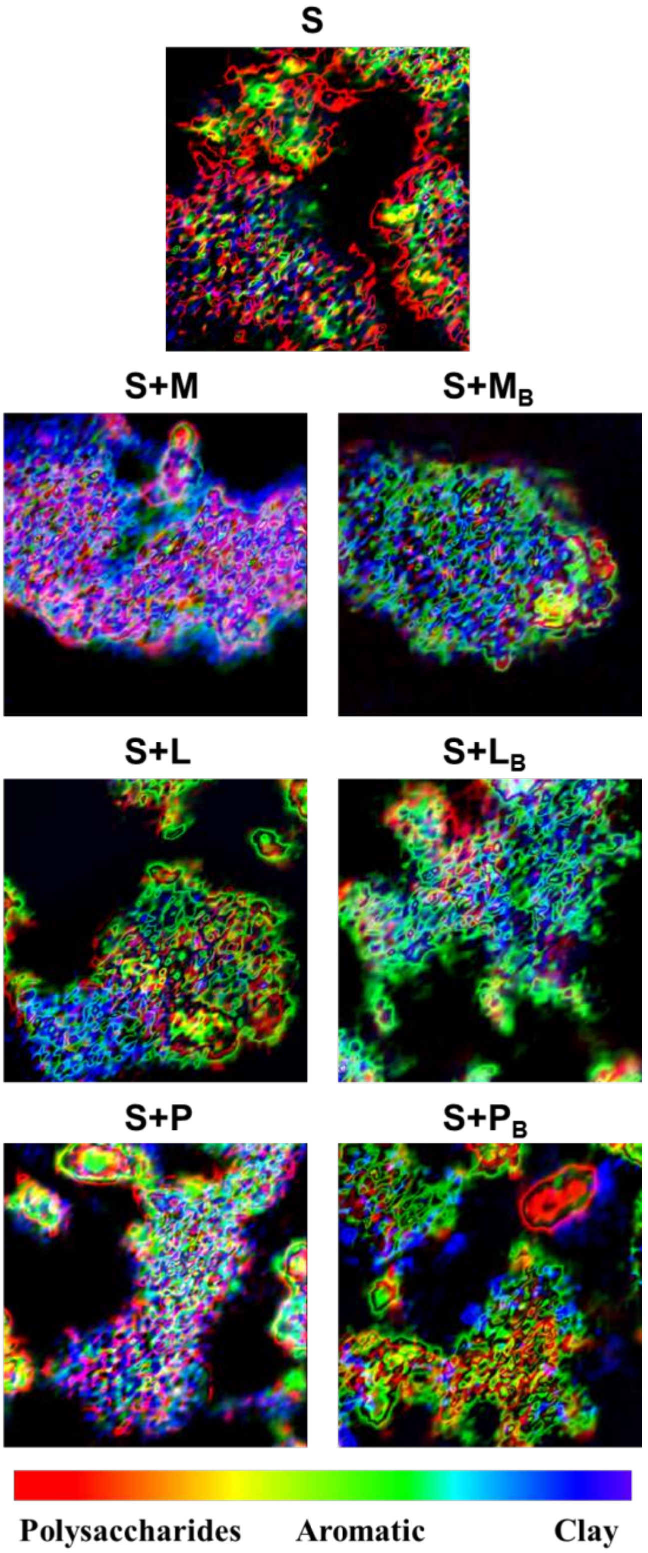

Figure S3. **Composite mapping of C speciation**. Red-Green-Blue (RGB) composites of spectral maps from soil microaggregates (<250 µm) isolated from non-amended soil (S) and soil amended with maize residue (S+M), leaf litter (S+L), peanut shell (S+P) or biochar (B) derived from those materials (S+M_B, S+L_B or S+P_B). The spectral maps were obtained using obtained by Fourier transform infrared spectroscopy (FTIR) coupled with optical microscopy.

Polysaccharides-C (1035 cm^{-1}) is red, aromatic-C (1600 cm^{-1}) is green and clay-OH (3630 cm^{-1}) is blue.

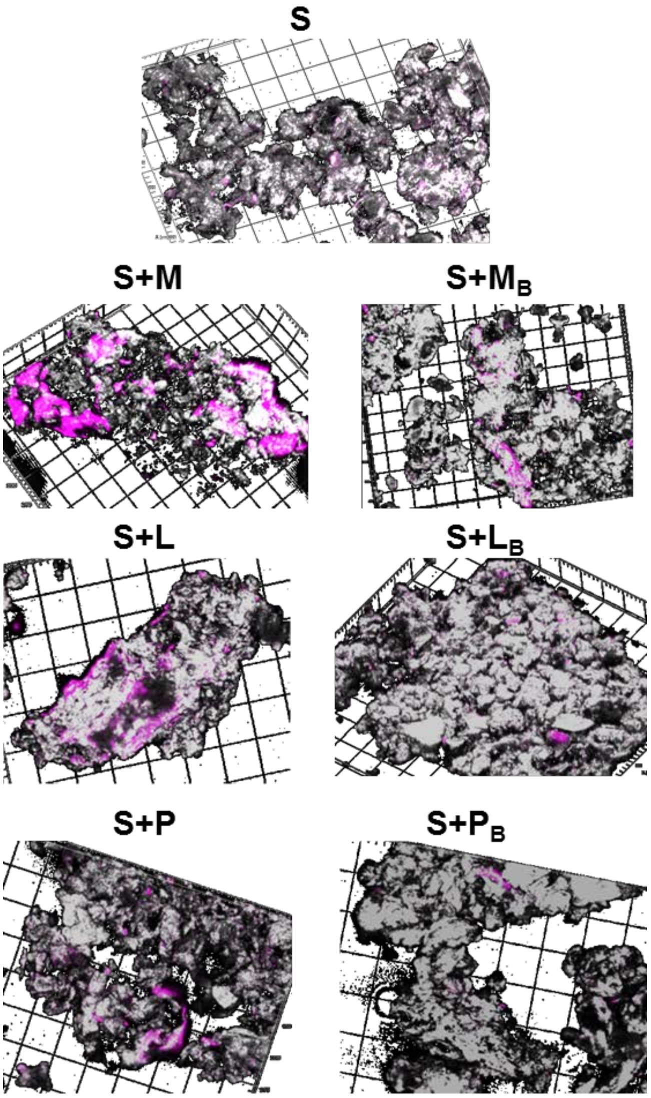

Figure S4. **3D-reconstruction of soil microaggregates**. Confocal laser scanning microscopy (CLSM) analysis visualizing autofluorescent organic matter (magenta) on microaggregates from non-amended soil (S) and soil amended with maize residue (S+M), leaf litter (S+L), peanut shell (S+P) or biochar (B) derived from those materials (S+M_B, S+L_B or S+P_B). Excitation with a 375 nm laser, emission captured between 455–500 nm. Surface profiling of the microaggregate was obtained by three-dimensional reconstruction of the z-series image stacks.

Table S1. **Relationship between carbon species**. Relationships, slopes and R-squared (R^2), between the amount of clay (absorbance at 3695 cm^{-1}), polysaccharides-C (absorbance at 1300 cm^{-1}), aromatic-C (absorbance at 1600 cm^{-1}) or aliphatic-C (absorbance at 2922 cm^{-1}) obtained from linear regression analyses. The coefficients are average and standard deviation values obtained from the regression analyses performed for three independent microaggregates. Treatments not connected by the same letter are significantly different ($p<0.05$) within the same column.

	**Polysaccharides-C/Clay**		**Aromatic-C/Clay**		**Aliphatic-C/Clay**	
	**Slope**	**R^2**	**Slope**	**R^2**	**Slope**	**R^2**
**S**	0.90 ± 0.10[a]	0.72 ± 0.08[a]	0.91 ± 0.04[a]	0.85 ± 0.05[a]	0.95 ± 0.09[ab]	0.89 ± 0.07[a]
**S+M**	1.23 ± 0.01[a]	0.77 ± 0.04[a]	1.08 ± 0.06[a]	0.82 ± 0.10[a]	0.80 ± 0.06[a]	0.75 ± 0.05[a]
**S+L**	0.91 ± 0.21[a]	0.52 ± 0.04[b]	0.96 ± 0.29[a]	0.55 ± 0.07[b]	1.15 ± 0.10[b]	0.64 ± 0.05[b]
**S+P**	0.95 ± 0.02[a]	0.50 ± 0.07[b]	0.82 ± 0.16[a]	0.80 ± 0.08[a]	0.83 ± 0.10[a]	0.88 ± 0.08[a]
**S+M_B**	1.02 ± 0.13[a]	0.68 ± 0.05[a]	0.93 ± 0.09[a]	0.75 ± 0.07[a]	0.91 ± 0.03[ab]	0.79 ± 0.04[ab]
**S+L_B**	0.99 ± 0.05[a]	0.77 ± 0.04[a]	0.98 ± 0.09[a]	0.90 ± 0.05[a]	0.94 ± 0.10[ab]	0.89 ± 0.05[a]
**S+P_B**	0.93 ± 0.10[a]	0.52 ± 0.05[b]	0.78 ± 0.10[a]	0.54 ± 0.07[b]	0.77 ± 0.10[a]	0.73 ± 0.04[b]

	**Aromatic-C/Polysaccharides-C**		**Aromatic-C/Aliphatic-C**		**Aliphatic-C/Polysaccharides-C**	
	**Slope**	**R^2**	**Slope**	**R^2**	**Slope**	**R^2**
**S**	0.94 ± 0.02[a]	0.92 ± 0.02[a]	0.97 ± 0.13[a]	0.86 ± 0.03[a]	0.84 ± 0.10[ab]	0.80 ± 0.05[a]
**S+M**	0.94 ± 0.12[a]	0.97 ± 0.03[a]	1.05 ± 0.06[a]	0.80 ± 0.08[a]	0.69 ± 0.12[ab]	0.73 ± 0.23[a]
**S+L**	0.91 ± 0.09[a]	0.93 ± 0.02[a]	1.05 ± 0.10[a]	0.91 ± 0.08[a]	0.82 ± 0.10[ab]	0.86 ± 0.06[a]
**S+P**	0.80 ± 0.12[a]	0.91 ± 0.02[a]	1.00 ± 0.07[a]	0.91 ± 0.04[a]	0.73 ± 0.08[ab]	0.84 ± 0.04[a]
**S+M_B**	0.92 ± 0.21[a]	0.85 ± 0.04[b]	0.91 ± 0.15[a]	0.88 ± 0.08[a]	0.94 ± 0.14[a]	0.88 ± 0.08[a]
**S+L_B**	0.89 ± 0.06[a]	0.83 ± 0.05[b]	1.02 ± 0.03[a]	0.90 ± 0.02[a]	0.76 ± 0.06[ab]	0.76 ± 0.03[a]
**S+P_B**	0.72 ± 0.11[a]	0.83 ± 0.04[b]	1.05 ± 0.12[a]	0.92 ± 0.08[a]	0.62 ± 0.08[b]	0.70 ± 0.08[a]
